# Cord pilot trial: update to randomised trial protocol

**DOI:** 10.1186/s13063-015-0936-2

**Published:** 2015-09-14

**Authors:** Lucy E. Bradshaw, Angela Pushpa-Rajah, Jon Dorling, Eleanor J. Mitchell, Lelia Duley, on behalf of the Cord Pilot Trial Collaborative Group

**Affiliations:** Nottingham Clinical Trials Unit (NCTU), Nottingham Health Science Partners, C Floor, South Block, Queens Medical Centre, Nottingham, NG7 2UH UK; Neonatal Unit, Queens Medical Centre, Nottingham, NG7 2UH UK

**Keywords:** Pilot, Randomised trial, Preterm birth, Umbilical cord clamping, Neonatal care at the bedside

## Abstract

**Background:**

The Cord Pilot Trial aimed to assess the feasibility of conducting a large UK randomised trial to compare the effects of alternative polices for timing of cord clamping (immediate within 20 seconds or deferred after at least 2 minutes) for very preterm birth before 32 weeks gestation. Initial recruitment was from March 2013 to February 2014, phase 2 was from March 2014 to February 2015. This paper updates the pilot trial protocol (Trials 15(1):258, 2014) and presents the changes for phase 2.

**Methods:**

An electronic randomisation system was introduced at three of the eight pilot sites. For follow-up of children, the Parent Report of Children’s Abilities – Revised (PARCA-R) will not be used. For children recruited to the trial during phase 2, follow-up at age 2 years (corrected for gestation at birth) will be by parent completed Ages and Stages Questionnaire (Squire J, Ages and Stages Questionnaires (ASQ), 2009) alone unless funds can be secured for the additional Bayley Scales of Infant Development III (Bayley N, Bayley Scales of Infant and Toddler Development, Third Edition. (Bayley-III), 2005) assessments. To assess accuracy of the cranial ultrasound diagnosis of intraventricular haemorrhage: (i) quality of the scans will be assessed using the British Society of Paediatric Radiology recommendations, and (ii) scan results will be confirmed by independent adjudication. Within and between adjudicator reliability will be assessed. In addition to the analyses planned to assess feasibility of the full trial based on data from the first year of recruitment, data on compliance and outcomes will be presented by allocated group for all women and babies recruited.

**Trial registration:**

ISRCTN21456601, registered on 28 February 2013.

## Update

### Background

The Cord Pilot Trial aimed to assess the feasibility of conducting a large randomised trial to compare the effects of alternative policies for timing of cord clamping (immediate or deferred) for very preterm birth (before 32 weeks gestation) in the UK. The protocol for this trial [1] presented methods for this feasibility assessment, which was based on recruitment from 1 March 2013 to 28 February 2014.

In October 2013, progress towards the targets for feasibility was considered sufficient to support application for funding for the full trial. This decision was supported by the independent Data Monitoring Committee (DMC) and by the independent Trial Steering Committee (TSC). The TSC advised that recruitment at the eight pilot sites should continue whilst funding for the full trial was sought, as this would maintain momentum and prevent loss of equipoise. To maximise efficiency of the planned full trial, pilot trial data would remain blind by allocated group and so would contribute to the sample size of the full trial. Recruitment into phase 2 of the pilot trial was commenced on 1 March 2014.

An application for funding of the Cord Trial was submitted to the National Institute for Health Research (NIHR) Health Technology Assessment (HTA) in the UK. The proposal was to recruit 2710 women and baby pairs from 45 UK sites, with a primary outcome of intraventricular haemorrhage (IVH). In February 2015, the application was rejected and recruitment to the Cord Pilot Trial, therefore, closed on 18 February 2015. This paper updates the pilot trial protocol and presents the changes for phase 2 of recruitment.

### Changes to methods during phase 2 of recruitment

#### Randomisation

For the full trial we planned to move to an electronic randomisation system, rather than continuing envelope-based randomisation as used in the Cord Pilot Trial. Therefore, an electronic randomisation system was tested at 3 of the pilot sites (from the 28th July 2014, 16 September 2014 and 23 September 2014 respectively). This was a secure web-based randomisation system maintained by Nottingham Clinical Trials Unit (NCTU), which could be accessed from a desktop computer or remotely using a hand-held device. The randomisation sequence for the electronic randomisation system was generated in the same way as the sequence for the envelope-based randomisation system.

Once a woman’s eligibility was checked and the necessary data entered into the electronic randomisation system, the woman was randomised. Sites received the allocated intervention directly from the web-based system. Details of the randomisation were emailed to the lead research nurse and the Principal Investigator at site, as well as to the trial manager and programming team at the NCTU. Trial stickers and Birth Record Sheets were available in a separate randomisation folder. The Birth Record Sheet could also be printed directly from the electronic randomisation system.

#### Follow-up

For children recruited to the trial from 1 March 2014 onwards, follow-up assessment at age 2 years (corrected for gestation at birth) is by parent completed Ages and Stages Questionnaire (ASQ [2]). This is sent by post, with a stamped addressed envelope to return the completed questionnaire. The Bayley Scales of Infant Development III (Bayley-III [3]) neurodevelopment assessment will be conducted for these children only if sufficient resources can be secured.

We had planned to use the Parent Report of Children’s Abilities – Revised (PARCA-R [4]) questionnaire for assessment of children in the Cord Pilot Trial. A change to the protocol for the pilot trial is that this will not be used, nor will it be used for children recruited in phase 2.

For women we cannot contact for the 1-year and 2-year follow-up, a letter will be sent to the women’s general practitioner (GP) asking them to check if the women’s contact details have changed or they have moved to a different practice.

For families that remain uncontactable at 2 years, a health status questionnaire asking about neurodevelopment will be sent to the Principal Investigator to complete based on the medical records at the child’s 2-year assessment.

### Assessment of brain injury from cranial ultrasound scans

To assess the accuracy and completeness of the diagnosis of intraventricular haemorrhage based on cranial ultrasound: (i) quality of the scans will be assessed, and (ii) scan results will be confirmed by independent adjudication. Quality of scans will be assessed against the British Society of Paediatric Radiology recommendations [5]. Adjudication will be by trained neonatologists and paediatric radiologists, using standard criteria. If there is disagreement between the adjudication assessment and the site scan report, a second adjudicator will be asked to review the scan images. Within and between adjudicator reliability will be assessed.

### Statistical analyses

In addition to the analyses planned to assess feasibility of the full trial based on data from the first year of recruitment, data on characteristics at trial entry, compliance with the allocated intervention and clinical outcome will be presented by allocated group for all women and babies recruited. The pilot trial was not designed to test a clinical hypothesis, but as intraventricular haemorrhage is the primary outcome for the planned full trial, this will be the main comparison for this analysis with the difference between groups reported with 95 % confidence intervals (CI). All other comparisons will be secondary analyses. For the analysis of multiple births, outcome will be assessed for total babies. Where appropriate, results will be presented as relative risk (RR) with 95 % CI, risk difference (RD) with 95 % CI, number of events prevented (compared with immediate clamping) per 1000 women with 95 % CI, or number needed to treat (NNT). Full details of the analysis are described in the Statistical Analysis Plan, which will be finalised before the randomisation allocation codes are released.

Analysis and reporting of the study will be in four stages:

(i) assessment of feasibility based on recruitment from 1 March 2013 to 28 February 2014
(ii) between group comparison of outcome at hospital discharge for all women and babies recruited
(iii) between group comparison of outcome at 1 year for all women recruited
(iv) between group comparison of outcome at age 2 years (corrected for gestation at birth) for all babies recruited

## Ethics approval

Approval for this study was granted by the Nottingham 2 Research Ethics Committee (NRES reference 12/EM/0283). All participants gave written informed consent or oral assent prior to randomisation. Further information on the consent pathways in the trial are provided in the full protocol paper [1].

## Abbreviations

ASQ: Ages and Stages Questionnaire
Bayley-III: Bayley Scales of Infant Development III
CI: confidence interval
DMC: Data Monitoring Committee
GP: general practitioner
HTA: Health Technology Assessment
IVH: intraventricular haemorrhage
NCTU: Nottingham Clinical Trials Unit
NIHR: National Institute of Health Research
NNT: number needed to treat
PARCA-R: Parent Report of Children’s Abilities –- Revised
RD: risk difference
RR: relative risk
TSC: Trial Steering Committee
